# Enhancing power density and cycle life of NMC811 battery cathodes *via* combined dense calendering and laser patterning

**DOI:** 10.1039/d5ee06773a

**Published:** 2026-02-04

**Authors:** Kumar Raju, Stephen W. T. Price, Alice J. Merryweather, Aleksandar Radić, May Ching Lai, Debashis Tripathy, Daniel Lorden, Edward Saunders, Israel Temprano, Sulki Park, Caterina Ducati, Akshay Rao, Angkur Shaikeea, Clare P. Grey, Michael De Volder

**Affiliations:** a Department of Engineering, University of Cambridge 17 Charles Babbage Road Cambridge CB3 0FS UK kr516@cam.ac.uk mfld2@cam.ac.uk; b The Faraday Institution, Quad One, Harwell Science and Innovation Campus Didcot OX11 0RA UK; c Finden Limited, Rutherford Appleton Laboratory Building R71 Harwell Oxford OX11 0Q UK; d Department of Materials Science and Engineering, The University of Sheffield Sheffield S1 3DJ UK; e Cavendish Laboratory, University of Cambridge J.J. Thomson Avenue Cambridge CB3 0US UK; f Yusuf Hamied Department of Chemistry, University of Cambridge Lensfield Road Cambridge CB2 1EW UK; g Illumion Ltd, Maxwell Centre J.J. Thomson Ave Cambridge CB3 0HE UK; h Department of Materials Science and Metallurgy, University of Cambridge Cambridge CB3 0FS UK; i School of Semiconductor and Chemical Engineering, Jeonbuk National University 567 Baekje-daero, Deokjin-gu Jeonju-si 54896 Republic of Korea

## Abstract

The charging time of Li-ion batteries is an important bottleneck in the wider adoption of electric vehicles (EVs). A common strategy to improve the rate performance is improving ion transport by patterning the electrode. However, these patterning methods usually increase the electrode porosity, thereby decreasing the volumetric energy density. In this work, we leverage the ability of Single Crystal LiNi_0.8_Mn_0.1_Co_0.1_O_2_ (SC-NMC811) electrodes to be calendered to higher packing densities than traditional cathodes, which then allows to offset additional porosity introduced by electrode patterning. We calendar SC-NMC811 electrodes to a 25% porosity and then introduce hole patterns spaced 100 to 600 µm apart using laser processing with a goal to maintain average porosities below 30%. As expected, we found systematic improvements in the rate performance with increasing hole density and used *operando* charge photometry to explore the limits of mass transport in the regions surrounding the holes but interestingly, we also observe improved capacity retention when using patterned electrodes. We found that there is less cathode lattice oxygen loss when using patterned cathodes, this in turn reduces transition metal shuttling reduces anode solid electrolyte interphase (SEI) impedance growth. We demonstrated a reduction in oxygen loss by both electron energy loss spectroscopy (EELS) mapping, X-ray diffraction (XRD) mapping and X-ray diffraction computed tomography (XRD-CT). Overall, SC-NMC811 electrode's ability to withstand over-calendering offers the opportunity to introduce laser patterned holes while maintaining the average porosity below 30%. This increases both the rate performance and longevity of the electrodes.

## Abstract 2

Over-calendering combined with laser patterning of battery cathodes allows for increased rate performance and lifetime without compromising volumetric energy density.

## Graphical abstract

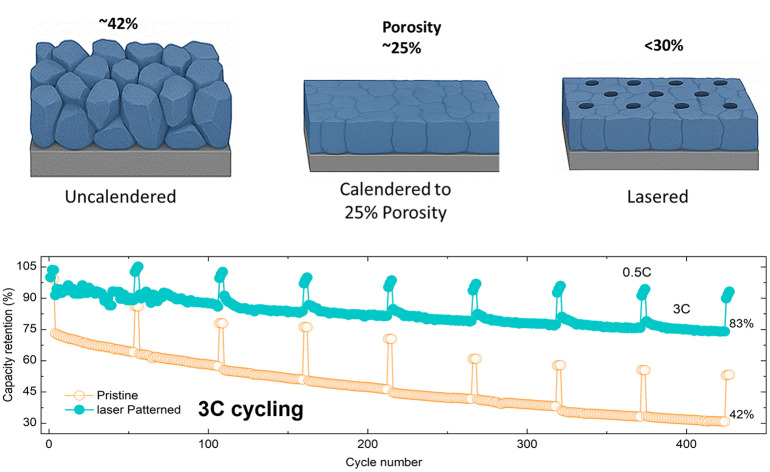

**Broader context**

Developing batteries with increased energy and power densities are key for the global net-zero transition. However, achieving fast charging without compromising energy density or lifetime remains a major bottleneck. Conventional methods to improve ion transport often involve making electrodes thinner or more porous which lowers energy per volume, while dense electrodes tend to degrade faster at high charging rates. Electrode patterning has been explored to improve power density, but most patterned electrodes exhibit porosities above 50%, far exceeding the commercial target of 35% or less. Our approach overcomes this limitation by calendering single crystal NMC811 cathodes densely to a porosity of 25% to offset additional porosity introduced judiciously by laser patterning to maintain the overall porosity below 30%. These dense patterned electrodes ensure high energy per volume while the laser channels enable fast charging without compromising lifetime. This work both introduces a new strategy for electrode design and unravels the processes that lead to reduced capacity loss in the proposed electrodes.

## Introduction

The development of electric vehicles is a key technology toward achieving net-zero emission targets. However, the relatively long charging times of Li-ion batteries (LIBs) have contributed to so called “range anxiety” and remain a bottle neck in the wider adoption of EVs. Aggressive targets have been set to achieve an 80% charge in 10 minutes to address this issue.^1,2^ Achieving fast charging is challenging because of the intrinsic kinetics of Li-ion batteries as well as issues such as heat generation and accelerated degradation taking place at high rate. Fast charging is sometimes pursued by using either very porous or ultra-thin electrodes to overcome Li transport limitations, but these approaches lead to poor energy per volume and weight densities.^3,4^ In thick electrodes, charge transport is primarily limited by lithium-ion diffusion, leading to concentration polarisation at high-rate, which causes preferential use of the material located closest to the separator membrane.^5,6^ Laser structuring of electrodes has been studied for over 15 years to enhance ion transport in battery electrodes.^7–11^ Lasers have been used to creates grooves and hole patterns mainly in anodes (graphite),^12–15^ but also in cathodes (LFP, LCO, LTO and NMCxyz).^7,8,16–19^ The improvements in rate performance in these patterned electrodes are largely credited to enhancements in ion transport (reducing tortuosity, and increasing porosity), mitigating state of charge (SoC) gradients over the thickness of the electrode. Further, it has been shown that laser patterning of anodes can mitigate anode degradation processes such as Li plating.^13^

**Fig. 1:**
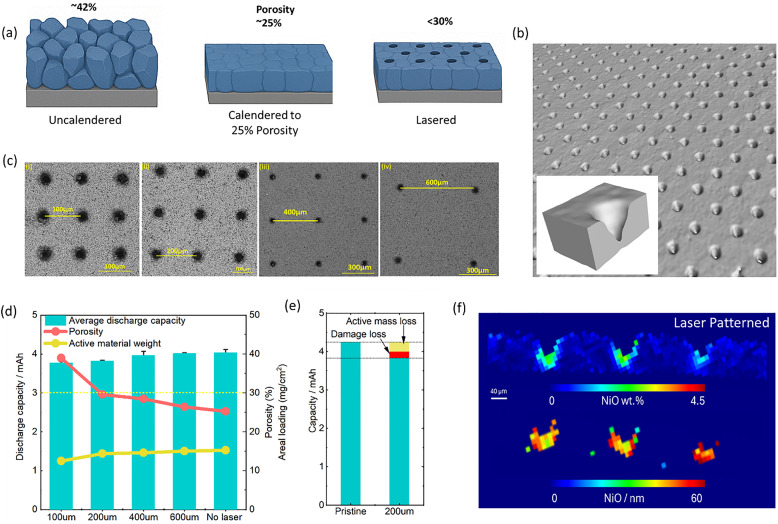
(a) Schematic of using over-calendering to offset laser-induced porosity in single crystal NMC811 electrodes. (b) X-ray μCT images of the laser-patterned electrode, with inset showing a magnified view of a laser patterned hole. (c) SEM images of laser patterned electrodes with different patterning distances ranging from 100–600 µm spacing. (d) Comparison of the average discharge capacities of a full cell at the 3rd formation cycle (C/20) with an error bar depicted the variability from two or more cells alongside the porosity and active material weight. (e) The capacity loss is broken down into loss of active material that is removed by the laser and loss from laser-induced damage on the surface (data for 200 µm spaced holes). (f) XRD-CT image showing NiO (wt%) distribution in the patterned holes.

While impressive, work reported in existing publications often conduct laser patterning on un-calendered electrodes, resulting in typical porosities of 45% to 50%, which are high for practical applications. Here we build on a recently made discovery that single crystal NMC811 cathodes can be calendered to much higher densities than classic poly-crystalline particles.^20^ As depicted in Fig. 1a, the starting point of our research is to calender our electrode to 25% porosity (*i.e.*, denser than the typical 30 to 35% porosity targeted in commercial electrodes) and then introduce limited amounts of laser patterns to operate at average porosities of 30% or less which is equal to or better than commercial practices.^21^ Despite this high density, these electrodes show excellent rate performance and increased cycling stability.

NMC811 cathodes are popular in automotive applications because of their high gravimetric energy densities of up to 250 Wh kg^−1^ in commercial batteries.^22,23^ However, Ni-rich layered cathodes tend to suffer from reduced cycle life compared to their low-Ni NMC alternatives, which is often accentuated at charge high rate. Here, we first show that laser patterning itself does not damage the cathode material substantially using micro computed tomography (μCT) and XRD-CT.^24,25^ Second, we carry out cycling experiments using 6C ultra-fast charging protocols and show laser patterned batteries achieve 80% capacity retention over 500 cycles, whereas where standard cells fail after less than 50 cycles under the same conditions. Distribution of relaxation time (DRT) derived from the electrochemical impedance spectroscopy (EIS) data shows that this is linked to a rapid increase in SEI impedance on the anode. Using elemental analysis, we reveal that these anodes have a higher transition metal (TM) content, which is known to catalyse SEI formation.^26–29^ EELS mapping of the cathode surface as well as XRD mapping and XRD-CT show that the patterned cathodes show less oxygen loss, which helps explains the reduced TM dissolution. Overall, laser patterning is able to increase the rate performance of SC-NMC811 cathodes without compromising their porosity and it extends their lifetime, both of which are critical attributes for next generation EVs batteries.

## Results

Electrodes with areal loadings of 15 mg cm^−2^ comprising of 90 : 05 : 05 of SC-NMC811, PVDF binder and Super-P conductive additive are used throughout this paper. The electrodes are calendered to 25% porosity before laser structuring. A nanosecond pulsed Yb fibre laser is used to drill holes with different spacings of 100, 200, 400 and 600 µm in the cathode. All cells are cycled with an additive comprising 1.3 M LiPF_6_ in EC : EMC : DEC (3 : 5 : 2 by vol) with 9 : 1 wt% FEC, 0.5 wt% VC, and 0.2 wt% LiBF_4_ as additives throughout this work.

The schematic in Fig. 1a illustrates densely packed single crystals with a reduction of porosity (to 25%) in comparison to that found in an uncalendered electrode (approximately 42%); laser drilled holes in the dense electrode further create a porosity varying from 28–33% based on the hole distance. SEM and X-ray μCT images of the laser patterned SCNMC811 electrodes are provided in Fig. 1b and c, and analysis of the tapered hole geometry is provided in Fig. S1. Using a microbalance, the mass loss by laser patterning is measured and the average porosity is calculated. As shown in Fig. 1d, electrodes with same hole pattern show highly reproducible electrochemical behaviour, and 3rd formation cycle capacities vary by only 6.5% for 100 µm spacing and 5.4% for 200 µm spacing over the two or more cells, the coulombic efficiency (CE) evolution is also consistent across multiple cells and only minor cell to cell variation after the first formation cycle (Fig. S2). Despite dense calendering, close packed holes (100 µm) result in average porosities of over 40%, which is too high for many applications, further, their high porosity leads to capacity fluctuations during cycling, which may be due to the highly porous structure crumbling under the applied stack pressure and volume changes of the active material during cycling (see further). At 200 µm spacing and above, the average electrode porosity drops below our 30% target. Therefore, initial tests were carried out in half-cells with holes spaced 200 µm apart (Fig. S3). First, we carried out synchrotron XRD-CT scanning to check for any evidence of laser induced degradation or redeposition of material. Small NiO crystallites with diameters of approximately 40–50 nm are observed at the bottom of the lasered holes (Fig. 1f), and there are limited to no signs of a temperature affected zone in other parts of the laser patterned. This corroborates the limited ∼5.2% of capacity loss measured for 200 µm spaced holes, which includes both active material removal and the combination of both thermal surface degradation and possibly also capacity loss due mechanical defects such active material flaking off in the patterned holes (see Fig. 1e and Fig. S4).

**Fig. 2:**
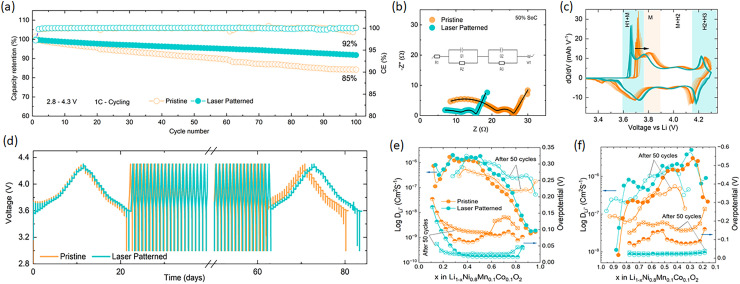
(a) Full cell cycling data at 1C using patterned and pristine cathodes paired with non–patterned graphite anodes. (b) EIS of patterned and non-patterned electrodes in a half cell with the inset showing the equivalent circuit used for EIS data fitting. (c) Differential capacity of patterned and non-patterned electrodes *versus* lithium metal. (d)–(f) GITT data of pristine and laser patterned electrode before and after 50 cycles at 0.5C.

Interestingly, as shown in Fig. 2a, the lasered electrodes (200 µm spacing) show improved capacity retention when cycled at 1C of 92% compared to 85% for pristine electrodes after 100 cycles, and 80% capacity retention at C/2 cycling after 400 cycles (Fig. S5) (longer term cycling is discussed further on). Next, EIS data taken after three formation at 50% SOC (Fig. 2b), shows a reduction of *R*_CT_ when patterning the electrode (see inset to Fig. 2b for definition of terms in the equivalent circuit model). These improvements in impedance are reflected in the rate performance, showing a 75% and 40% capacity retention at 3C and 5C respectively (Fig. S6). By our DRT analysis, the lasered electrode half-cell shows a reduced *R*_SEI_ = 4.4 Ω and *R*_CT_ = 5.5 Ω *vs. R*_SEI_ = 12.1 Ω and *R*_CT_ = 8.4 Ω in the pristine electrode half-cell.

As evidenced by the differential capacity (d*Q*/d*V*) curves in Fig. 2c and Fig. S7 there is a clear increase in polarisation in the pristine electrodes which is supressed in the laser patterned ones. Galvanostatic intermittent titration technique (GITT) was used to investigate differences in lithium diffusion and resistance as a function of SOC for pristine and laser-patterned electrodes before and after 50 cycles. As shown in Fig. 2d–f, significant improvements in Li ion diffusion (*D*_Li^+^_) are observed in the laser patterned electrodes (see SI Table S1 for detailed data). In particular, the overpotential of the pristine cells increases after cycling, Fig. 2(e) and (f), which is in agreement with the pronounced peak shifts in (d*Q*/d*V*) curves and will be discussed in more detail further on.

### Pattern optimisation

**Fig. 3:**
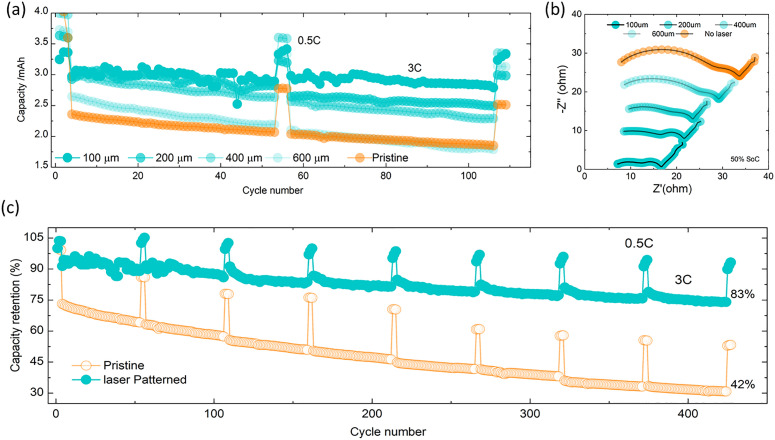
(a) Power capability of laser patterned electrodes for different distances of holes at 3C for 100 cycles with 0.5C recovery cycles at every 25 cycles. (b) EIS profiles of pristine and laser patterned electrodes after the formation cycles at different distances of holes. (c) Laser-patterned (100 µm spacing) and non-patterned NMC811 cycle stability at 3C cycling with 0.5C recovery cycles every 50-3C cycles.

The spacing of laser-drilled holes needs to be chosen judiciously to balance the amount of active material removal with increases in rate and cycling stability. To investigate this trade-off, which ultimately depends on the application's power *versus* energy density requirements, the four different hole spacings (100 µm, 200 µm, 400 µm, and 600 µm) were evaluated in full cells. Fig. 1d and Fig. S4 corroborate the capacity loss associated with material loss during the laser process. Although the charge–discharge capacity remains relatively high across all electrodes, 100 µm spaced holes lead to an average 6.4% drop in capacity compared to pristine electrode, and results in a battery porosity of over 40%, which is relatively high. Also, Fig. 3a shows that these highly porous electrodes yield fluctuations in capacities during the first 100 cycles, which might be due to crumbling of the relatively thin sections of battery materials between the holes and in combination with the effect of stack pressure and particles swelling and shrinking during cycling. From spacings of 200 µm and upwards, the overall porosity drops below 30%, but the impedance of the electrode grows with increasing spacing to charge transfer resistance (*R*_CT_) value of ∼5 Ω for a spacing of 100 µm to a value of 28 Ω for the unpatterned electrode. Fig. 2b and Fig. S8 show that, when analysed by DRT, an increase in hole density significantly reduces the SEI resistance (*R*_SEI_); the pristine electrode exhibited an *R*_SEI_ = 21.3 Ω, whereas the electrode with 100 µm spacing showed an *R*_SEI_ = 4.3 Ω. For spacings of 600 µm, there are no benefits in cycling performance with unpatterned electrodes as shown in Fig. 3a, which suggests that in our test conditions the optimal spacing is between 200 and 400 µm.

We have also investigated other patterns such a laser patterned lines (see Fig. S9), yet these do not lend themselves as well to high volumetric density electrodes. We have also measured the effect of different depths of electrode patterns (see Fig. S9) and verified that we can process other cathode materials such as LFP and LNO (see Fig. S10). Next, we examined the effect of laser structuring on battery cycling at 3C rate over 400 cycles. Fig. 3c demonstrates that under these conditions, laser patterned electrodes yield substantially higher capacity, which is a result of laser patterning providing better ion transport, as explored further below.

**Fig. 4:**
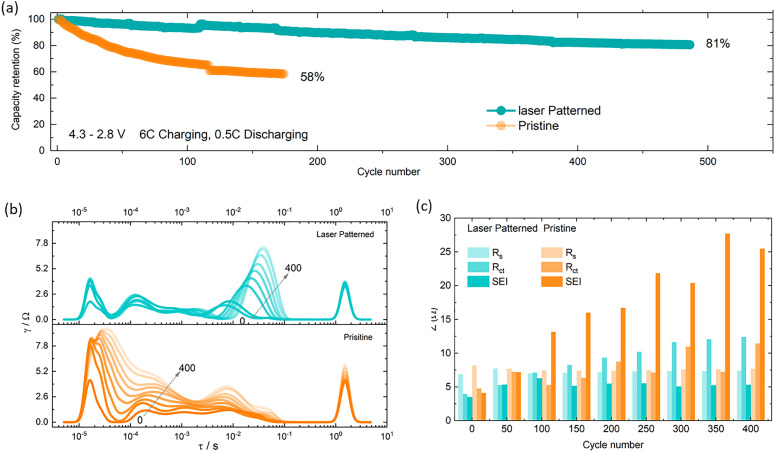
(a) Long term cycle stability of laser patterned and pristine electrode at 6C-charging/0.5C discharging. (b) and (c) distribution of relaxation time (DRT) derived from the EIS data measured at 50% SoC at every 50 cycles at 3C and the resistance values of *R*_s_, *R*_CT_ and *R*_SEI_ extracted from it.

Importantly, laser patterning also influences the lifetime of the battery. We found that the capacity retention is improved to 83% over 400 cycles for patterned electrodes, compared to 42% for pristine ones. Every 50 cycles at a 3C rate, 3 diagnostic cycles were run along with 0.5C at which point we carried out EIS measurements at 50% SOC to monitor impedance build-up (Fig. S11a and b). The pristine electrode showed a faster impedance build-up ((∼11 Ω) increase in *R*_CT_ per 50 cycles *versus* ∼2.5 Ω for patterned electrodes) which was further confirmed with a DRT analysis as shown in Fig. 4b and c. There is no significant changes observed in *R*_s_ (7–7.5 Ω) and *R*_SEI_ (4–5 Ω) values, and a small increase in *R*_CT_ values (∼2 Ω upsurge for each 50 cycles) of patterned electrode. However, the pristine upsurges both *R*_CT_ (4–12 Ω) and *R*_SEI_ (4–25 Ω), but no changes in *R*_s_ (∼7.5 Ω).

As set out in the introduction, fast charging is an increasingly important requirement for next generation EV batteries, yet most battery chemistries suffer from faster degradation under these conditions, especially when using high packing density electrodes. To further investigate the degradation trends from Fig. 3c, cells were tested using an asymmetric fast charging (6C), slow discharging (0.5C) protocol. As shown in Fig. 4a electrodes with 200 µm spacing achieved a capacity retention of 81% over 500 cycles, while under the same conditions, the pristine electrode capacity drops to 58% after only 200 cycles (Fig. 4a and Fig. S12). Similar to 3C cycling, EIS measurements were carried out at 50% SOC after every 50 cycles to monitor impedance build-up (Fig. S11(c) and (d)), the pristine electrode showing higher impedance build-up than the lasered electrode. The mechanism of this impedance build-up is discussed further below.

### Effect of laser patterning on ion transport

**Fig. 5:**
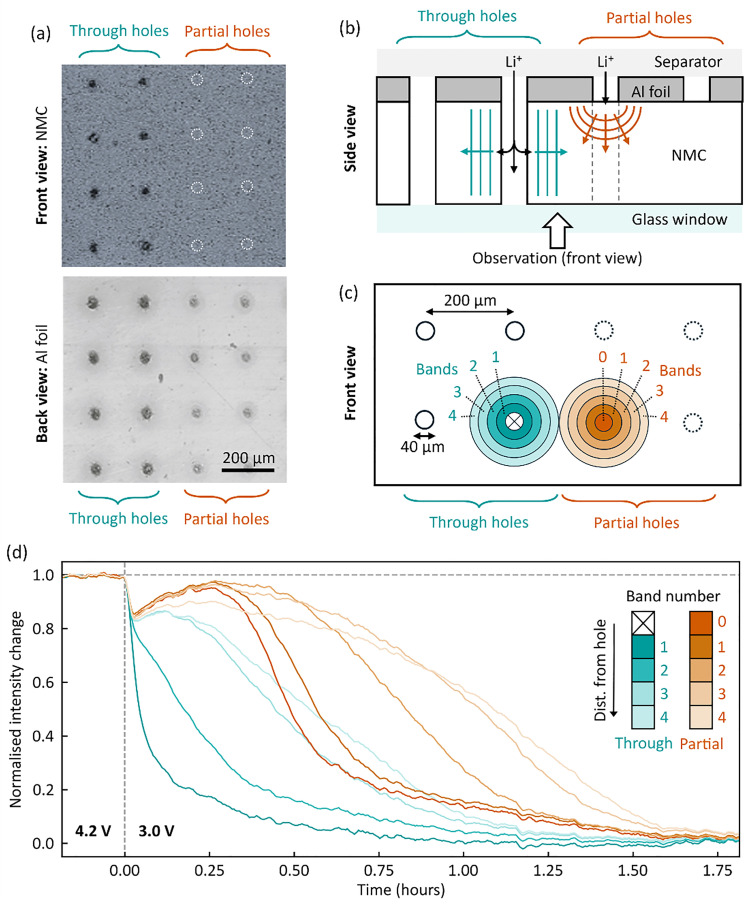
(a) A special electrode patterned with a mixture of ‘through holes’ (piercing NMC and current collector) and ‘partial holes’ (piercing current collector only), 40 µm diameter holes, 200 µm spacing. Upper image: Front view of NMC side, with dashed white circles indicating positions of partial holes. Lower image: Back view from Al foil side. (b) Schematic cross-sectional view of the electrode, as assembled in a windowed coin cell, with the active material facing away from the separator to enable observation of the electrode *via* a glass window. Arrows illustrate the different paths that Li-ions must take to lithiate the NMC in this geometry. (c) Schematic front view of the electrode, representing the field-of-view imaged by *operando* charge photometry. Solid-line and dashed-line circles indicate ‘through’ and ‘partial’ holes respectively. The area surrounding each hole is divided into concentric rings or ‘bands’ (external radius increasing by 20 µm for each subsequent band), illustrated in shades of blue (‘through’ hole) and orange (‘partial’ hole), (d) *operando* charge photometry data obtained during lithiation of the cell *via* an abrupt step between 4.2 V and 3.0 V (step occurring at 0 hours). The (normalised) intensity of reflected light is plotted for different regions of the electrode surface, matching the radial bands illustrated in panel (c). Lower values of intensity indicate more lithiated NMC.

To explore the effect of laser patterning on ion transport in the electrode, we performed charge photometry measurements (an *operando* imaging technique based optical scattering microscopy)^30–32^ to map the real-time lithiation following an abrupt potentiostatic step from 4.2 V to 3.0 V. An adapted electrode was prepared with a mixture of ‘through’ holes (penetrating both current collector and active material layers) and ‘partial’ holes (penetrating only the current collector) with a 200 µm spacing, as shown in Fig. 5a. The electrode was assembled in an optically-accessible coin cell with the current collector facing towards the separator, such that electrolyte access to the active material is only *via* the lasered holes (Fig. 5b).

Images were obtained of a region of the electrode surface containing both ‘through’ holes and ‘partial’ holes, throughout the lithiation. Charge photometry spatially-resolves changes in the intensity of light reflected by the electrode surface, where for NMC – lower intensity values indicate a higher local degree of lithiation.^31–33^ To interrogate the spatial dependence of lithiation rate, the electrode surface was divided into concentric rings or ‘bands’ at various distances from each hole type (external radius increasing by 20 µm for each subsequent band, schematic Fig. 5c), and the intensity response for each band is shown in Fig. 5d. For the ‘through’ holes, a clear radial dependence is observed as regions closer to the hole were seen to lithiate (decrease in intensity) before further away regions, indicative of lithium-ions diffusing radially outwards from the lasered channel (blue arrows, Fig. 5b).

A similar radial dependence was observed for the ‘partial’ holes and, importantly, a consistent 30–40 min time delay was observed compared to 'through’ holes. For example, ‘partial’-hole band 2 (spanning 40–60 µm from the hole centre) took ∼39 min longer than its ‘through’ hole counterpart to reach a normalised intensity change of 0.5, due to its longer ion transport pathway vertically through the electrode thickness (red arrows, Fig. 5b). (See SI section ‘charge photometry experiments’ for further discussion, including intensity line shapes). This observation clearly indicates that laser-patterned channels through the full thickness of active material layer (here ∼60 µm) offer improved rates of ion transport through the electrode thickness, which can contribute to improved performances at high cycling rates, especially above ∼2C, where mass transport on the timescales of 30–40 min would become directly limiting.

### Degradation mechanism

**Fig. 6:**
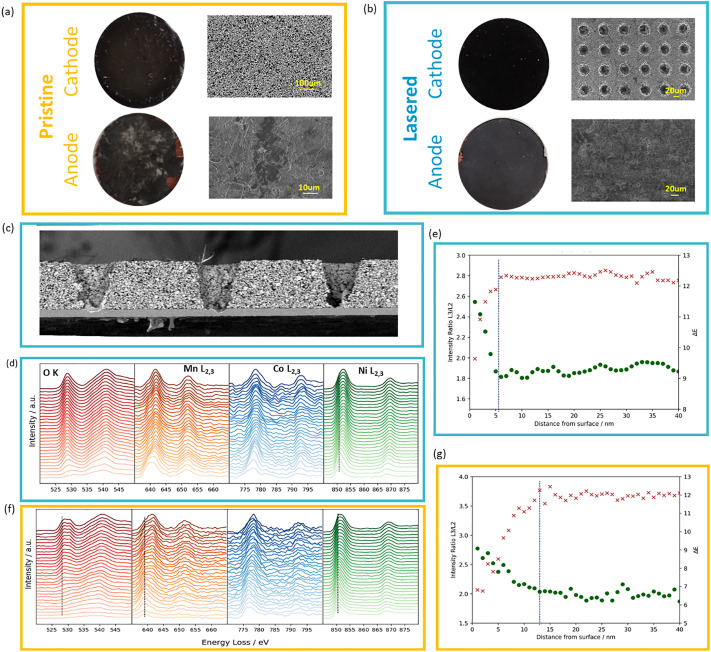
Photograph of cycled electrodes and their corresponding SEM images (a) and (b). (c) SEM cross-section image of the cycled patterned electrode. EELS mapping of the Ni, Co, Mn L edges and O K edge, and the intensity of Ni L edge analysed for electrodes cycled at 3C over 400 cycles. Laser patterned electrode (d) and (e) and pristine (f) and (g).

First, we looked at a high level at the mechanical integrity of patterned and pristine electrodes after cycling. Post-mortem digital images show a grey discoloration of anodes in cells without laser patterning, which is less prominent in cycled laser patterned electrodes (Fig. 6a and b), which may be an indication of more SEI decomposition products formation. The patterned electrodes show limited increase in thickness of 14 µm from the initial 75 µm after 400 cycles at 3C whereas unpatterned electrodes show thickness increase of 32 µm. Further, SEM analysis of the ion-milled cross-sections of the cycled electrode shows that the pore structure is largely preserved after high-rate cycling, and no obvious pore collapse or clogging is observed (Fig. 6c). Next, we will evaluate in more details the mechanism of lifetime extension in cells with patterned cathodes.

While laser patterning of graphite anodes has shown to improve lifetime at high C rates by reducing the occurrence of Li plating,^13^ the mechanism of increased stability when patterned cathodes observed in this work is less clear. An important degradation mechanism in NMC811 *vs.* graphite cells is linked to oxygen loss from the cathode surface, which leads to transition metal dissolution in the electrolyte, which in turn poisons the anode SEI.^34–36^ Increased SEI formation consumes the cell Li inventory, which is the main source of capacity loss in this battery chemistry.^37^ In what follows, we investigate if there are signs of reduced surface oxygen loss and transition metal dissolution in cells using laser patterned electrodes. First, we use EELS mapping to assess the thickness of the reduced surface layer of pristine and lasered NMC cathodes after 400 cycles at 3C. The line spectrum and intensity ratio plots (Ni, Mn, Co L3/L2 ratios) in Fig. 6d–g track elemental changes as a function of distance from the electrode surface. The main peaks of the Ni L3 edge and O K edge of pristine electrode showed a clear peak shift to higher energy levels over a thickness of approximately 15 nm (Fig. 6e), whereas in the laser-structured electrode this shift only occurs over a thickness of 4 nm (Fig. 6g), suggesting a thinner surface reduced layer and less oxygen loss. Similarly, the L3 peaks of Co and Mn also showed a more pronounced peak shift in pristine than lasered electrode (Fig. S13).

**Fig. 7:**
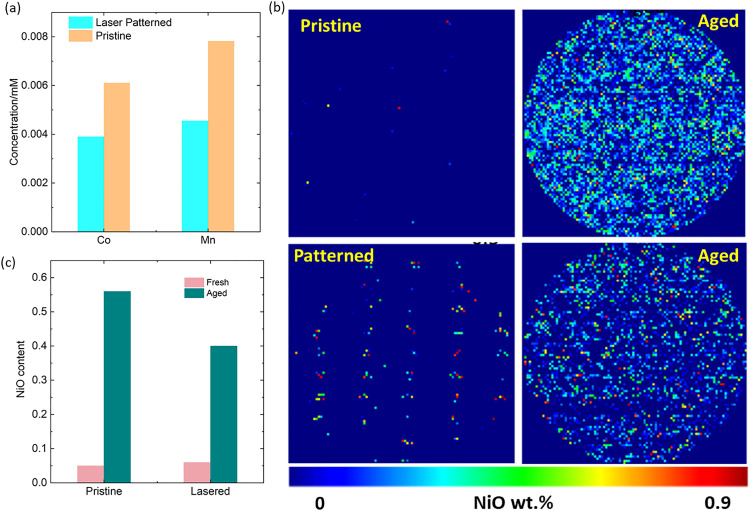
(a) MP-AES data of dissolution of transition metals on the graphite anode after the electrodes were cycled over 400 at 3C. (b) Rietveld refinement using XRD maps showing NiO phase distributions in both fresh and aged pristine and patterned electrodes. (c) Surface reduced layers (NiO) quantified by synchrotron XRD CT analysis of fresh and cycled cells with and without laser patterning.

While the absolute value of cathode capacity loss from these relatively thin reduced surface layers is moderate, the associated dissolution of TMs from the cathode surface into the electrolyte and their subsequent poisoning of the anode SEI is known to be a major cause of capacity loss.^34,37–39^ A first indication of this mechanism being at play, is the increase in SEI impedance as shown by DRT-EIS analysis in Fig. 4c. Further, to verify the increase in transition metal cross-over on the anode, we digested anodes cycled 300 times and carried out elemental analysis by MP-AES. As shown in Fig. 7a, this data shows a reduction of 36% Co and 42% Mn in TM content on the anode, as a result of the laser patterning.

Finally, the effect of laser patterning on the formation of surface reduced layers was quantified by synchrotron XRD mapping analysis of fresh and cycled cells with and without laser patterning (Fig. 7b). For this, the NiO content within the laser patterned holes was masked (*i.e.*, was not included in the total NiO content), which yields a baseline of 0.04 wt% NiO in pristine electrodes and 0.05 wt% in laser patterned ones. (the fitted error in these values is 0.04 wt%, *i.e.*, within error no NiO is present on the surface of either pristine cathodes). This masking is used to differentiate between the NiO introduced by laser patterning during the electrode manufacturing and by surface reduction during cycling. Analysis of aged samples shows 0.54 wt% NiO content in pristine electrodes compared to 0.4 wt% in laser patterned ones (Fig. 7c). This again suggests more oxygen loss from the surface of unpatterned cathodes. We suspect that this is a result of a more uniform use of the cathode material in lasered electrodes, and as a result less electrochemical stressing of certain parts of the electrodes, supressing cathode degradation processes as observed above.

## Conclusions

In this work, we calender single crystal NMC811 to 25% porosity, and then introduce limited amounts of laser patterned holes, keeping the overall porosity below 30% to retain good volumetric performance. Single crystal particles lend themselves to this process as they are able to withstand higher calendering densities, but also because often relatively large crystals can lead to slow kinetics, which means that patterning results in important enhancements in rate performance. We demonstrate these benefits by porosity analysis, photometry and rate tests. Interestingly we also observe increases in capacity retention when using patterned cathodes. Using both EELS mapping and synchrotron XRD mapping, we demonstrate that laser patterning suppresses oxygen loss from the cathode surface. This in turn leads to less TM dissolution and shuttling to the anode, as shown by MP-AES. We observe reduced SEI formation, as shown by DRT-EIS, and leads to less Li-inventory loss, corroborating the observed increases in capacity retention. Overall, this study shows that a judicious introduction of laser patterned holes in electrodes can lead to both improvements in power density and lifetime without substantial sacrifices in volumetric performance.

## Experimental procedure

### Electrode preparation

A single-crystal (SC) LiNi_0.8_Mn_0.1_Co_0.1_O_2_ (SCNMC 811) electrode sheet with a mass loading of 15 mg cm^−2^ was procured from LiFun. The electrode sheet was calendered to a porosity of 25%. The areal capacity was calculated to be ∼2.77 mAh cm^−2^, based on the SCNMC 811 cathode capacity of 185 mAh g^−1^. The graphite anode slurry was prepared by mixing 91.7 wt% artificial graphite (MTI), 6 wt% polyvinylidene fluoride (PVDF), 2 wt% conductive carbon, and 0.3 wt% oxalic acid using *N*-methyl-2-pyrrolidone (NMP) as the solvent. The slurry was uniformly cast onto a 20 µm copper foil and dried. The resulting anode sheet has a mass loading of ∼9 mg cm^−2^, corresponding to an areal capacity of ∼3.06 mAh cm^−2^, based on the graphite anode capacity of 340 mAh g^−1^.

### Laser structuring

The 3D vertical pores were created on the calendered electrode using an SPI G3 20 W nanosecond pulsed Yb fibre laser with a wavelength of 1065 nm. The laser parameters were optimized for uniform structuring and set as follows: average power of 8 W, pulse frequency of 25 kHz, scanning speed of 3000 mm s^−1^, and a pulse duration of 0.5 s. The laser parameters were carefully controlled to ensure not pierce the aluminium current collector for all the electrochemical analysis except the optical scattering measurements.

### Electrochemical cell assembly and protocols

2032-Type coin cells (CES, Cambridge) were assembled within an Argon-filled glovebox. Half-cells featured a 13 mm cathode, 15 mm lithium metal (Hohsen), and a 19 mm Celgard separator soaked in 1.3 M LiPF6 in EC : EMC : DEC (3 : 5 : 2 by Vol) with 9 : 1 wt% FEC, 0.5 wt% VC, and 0.2 wt% LiBF4 (E-lyte Germany). Full cells contained a 14 mm cathode, 15 mm graphite anode, and a 260 µm thick GF/B grade glass fibre separator soaked in 100 µL 1.3 M LiPF6 in EC : EMC : DEC (3 : 5 : 2 by Vol) with 9 : 1 wt% FEC, 0.5 wt% VC, and 0.2 wt% LiBF_4_ (E-lyte Germany). After assembly, half cells underwent three formation cycles (CCCV, C/20, 3–4.3 V). Full cells were tap-charged to 1.5 V and rested for 10 h, followed by three formation cycles at C/20. Rate capability and cycle stability were assessed after these conditioning steps. All the electrochemical measurements were conducted in a climate chamber set at 26 °C. Electrochemical impedance spectroscopy (EIS) was carried out with the Biologic BCS 805 Series at 3.8 V and 10 mV amplitude, and the scanning frequency spanned from 10 kHz to 10 mHz. DRT analysis was carried out in custom python scripts using the open-source software, pyDRTtools. EIS data below 0.1 Hz was trimmed for the DRT analysis, and the resulting distorted diffusion peak was not further considered. For the pyDRTtools fitting,^38^ Gaussian radial basis functions were centred at (2π*fk*)^−1^, where *fk* are the measured frequencies. The FWHM of the basic functions were set to twice the ln *τ* spacing of their centres. Lumped series resistances and inductances were included in the parameter fitting. The DRT fit was calculated from both the real and imaginary parts of the data using Tikhonov regularisation with a penalty term based on the first derivative of the DRT. A regularisation parameter of *λ* = 1*e*^−3^ was selected for all fittings. Peak quantification was then performed by fitting skewed gaussians, their area being used to calculate component impedance contributions.^39^ In a typical spectrum, 4–5 major peaks were observed at time constants beyond the discounted diffusion associated feature. From the literature, such peaks are typically attributed to the processes of stray/interfacial impedances, SEI, CEI, cathode *R*_CT_, anode *R*_CT_, and diffusive processes (from fastest to slowest time constant).^40^ However, we could not deconvolute these processes for all the measured spectra. Therefore, we chose to sum our fastest 2 peaks and label them as SEI (containing contributions from all the stray, SEI, and CEI impedances), and to attribute the remaining non-diffusive peaks to *R*_CT_, as other authors have done.^41^

### Charge photometry collection, processing, and analysis

The electrode was assembled in an optically accessible coin cell, with the NMC layer facing the glass window. Cell assembly was as described above for half cells, except that a *n* additional disk of fine stainless-steel mesh was included between the electrode and the separator to ensure good electronic contact to the current collector in this geometry.

The cell was first formed *via* normal CCCV cycling protocols. For the potential step protocol, A voltage of 4.2 V was first applied for 4 hours to delithiate the electrode, followed by a 30 min OCV, and then a potentiostatic step to 3.0 V for 4 hours.

Charge photometry was conducted using a wider field-of-view adaptation of a previously reported microscope setup.^30,32^ This consisted of a custom-built inverted wide-field microscope fitted with an air objective (20×/NA0.4, TU Plan EPI ELWD, Nikon) with wide-field illumination (740 nm, Thorlabs fiber-coupled LED, M740F2). Polarisation optics imaged the sample onto a CMOS camera (FLIR, Grashopper3 GigE, GS3-PGE-91S6M-C). The sample was mounted with a custom cell holder onto an inverted microscope stage (Mad City Labs, MicroStage and Nano ZL500). The microscopy and electrochemistry were controlled concurrently using a custom Python-based interface. We note that this microscope setup formed a prototype for a charge photometry instrument (‘illumionONE’) which has subsequently been commercially developed by ‘illumion’.

Charge photometry images were analysed by finding the centres of each hole and defining bands around each such that each subsequent band has an outer radius 20 µm larger than the previous, thus separating the visible active material into radial groups. We then track the mean intensity within each band as a function of time to examine the radial dependence of lithiation.

### XRD mapping and XRD-CT collection, processing, and analysis

XRD data was measured using beamline P07 (EH2) at PETRA III, DESY, Hamburg. The beam energy was 73.4 keV (0.169 Å), focussed to 2 × 20 µm (vertical × horizontal). Data was collected using an Eiger2 CdTe 4 M hybrid pixel detector at 500 mm sample-detector distance and at 100 Hz. The detector position, distance and orientation were calibrated using a CeO_2_ reference standard (NIST, 99.9%, <5 µm). The samples were mounted perpendicular to the beam path for XRD mapping, and raster scanned with 10 µm horizontal and vertical step size. The samples were then wrapped around a 1 mm diameter polyimide tube for XRD-CT data collection. The XRD-CT measurement had a horizontal rotation axis and were scanned with continuous translation (vertical) steps of 10 µm in a zig-zag configuration, over a 0–360° rotation range using steps of 0.9°. A beam monitor diode was used for sample absorption correction. The resulting pixel size was also 10 × 10 µm to match that of the XRD mapping. The raw XRD data (2D images) were integrated using PyFAI^42^ to generate the maps. XRD-CT data processing and reconstruction by filtered back projection was performed using in-house Matlab scripts. Data refinement was performed using Topas V7^43^ with Al (ICSD 18839), NiO (ICSD 112324) and LiNiO_2_ (ICSD 44263 – representing the *R*3̄*m* NMC811 structure) as input models.

### EELS mapping

Cross-sectional lamellae were prepared to electron-transparent thicknesses (<100 nm) in a FEI Helios NanoLab DualBeam focused ion beam-scanning electron microscope (FIB–SEM, Thermo Fisher Scientific) following standard protocols. Lamellae were transferred directly to a Thermo Fisher Spectra 300 TEM operated at 300 kV, minimising air exposure to <2 min.

EELS spectrum imaging was performed with a dwell time of 50 ms per pixel and a pixel size of 1 nm. The beam current was ∼130 pA, and spectra were collected with a collection semi-angle of 17 mrad. DualEELS acquisition at 0.3 eV per channel dispersion was used to record the low-loss (including the zero-loss peak) and core-loss spectra simultaneously at each probe position. The energy resolution, estimated from the full width at half maximum of the zero-loss peak, was ∼1 eV. Acquisition parameters were optimised to balance energy resolution and signal-to-noise while avoiding measurable beam damage to NMC811. Resulting spectrum images were processed in HyperSpy.

## Author contributions

Kumar Raju: conceptualization, data curation, formal analysis, investigation, methodology, project administration, writing – original draft, writing – review & editing. Stephen WT Price: data curation, formal analysis. Alice J. Merryweather: data curation, formal analysis, writing – review & editing. Aleksandar Radic: data curation, formal analysis. May Ching Lai: data curation, formal analysis. Debashis Tripathy: formal analysis. Daniel Lorden: formal analysis. Edward Saunders formal analysis. Israel Temprano: formal analysis. Sulki Park: formal analysis. Caterina Ducati: resources, writing – review & editing. Akshay Rao: resources, writing – review & editing. Angkur Shaikeea: data curation. Clare P Grey: writing – review & editing. Michael De Volder: conceptualization, data curation, formal analysis, funding acquisition, investigation, project administration, supervision, writing – review & editing.

## Conflicts of interest

Alice J. Merryweather, Akshay Rao and Clare P. Grey are founders of Illumion, Ltd.

## Supplementary Material

EE-019-D5EE06773A-s001

## Data Availability

The data supporting the findings of this study are available from the authors upon reasonable request. Supplementary information (SI) is available. See DOI: https://doi.org/10.1039/d5ee06773a.
