# Supplementary material for: Enhancing power density and cycle life of NMC811 battery cathodes via combined dense calendering and laser patterning
Source: Energy Environ Sci. 2026 Feb 4;19(4):1341–51. doi: 10.1039/d5ee06773a (PMC12875191; doi:10.1039/d5ee06773a)
Supplement: EE-019-D5EE06773A-s001 [file EE-019-D5EE06773A-s001.pdf]

Supplementary Information (SI) for Energy & Environmental Science.
This journal is © The Royal Society of Chemistry 2026

Supporting Information

Enhancing Power Density and Cycle Life of NMC811 Battery Cathodes via Combined Dense Calendering and Laser Patterning

Kumar Raju[ab*], Stephen WT Price[bcd], Alice J. Merryweather[befg], Aleksandar Radić[e], May Ching Lai[bh], Debashis Tripathy[bf], Daniel Lorden[bd], Edward Saunders[af], Israel Temprano[f], Sulki Park[i], Caterina Ducati[bh], Akshay Rao[be], Angkur Shaikeea[a], Clare P Grey[bf], Michael De Volder[ab*]

[a]*Department of Engineering, University of Cambridge, 17 Charles Babbage Road, Cambridge, CB3 0FS, UK*
[b]*The Faraday Institution, Quad One, Harwell Science and Innovation Campus, OX11 0RA, Didcot, UK*
[c]*Finden Limited, Building R71, Rutherford Appleton Laboratory, Harwell, Oxford, OX11 0Q, UK*
[d]*Department of Materials Science and Engineering, The University of Sheffield, Sheffield, S1 3DJ, UK*
[e]*Cavendish Laboratory, University of Cambridge, J.J. Thomson Avenue, Cambridge, CB3 0US, UK*
[f]*Yusuf Hamied Department of Chemistry, University of Cambridge, Lensfield Road, CB2 1EW, Cambridge, UK*
[g]*Illumion Ltd., Maxwell Centre, J.J. Thomson Ave, Cambridge, CB3 0HE, UK*
[h]*Department of Materials Science and Metallurgy, University of Cambridge, Cambridge, CB3 0FS, UK*
[i]*School of Semiconductor and Chemical Engineering, Jeonbuk National University, 567 Baekje-daero, Deokjin-gu, Jeonju-si 54896, Republic of Korea*

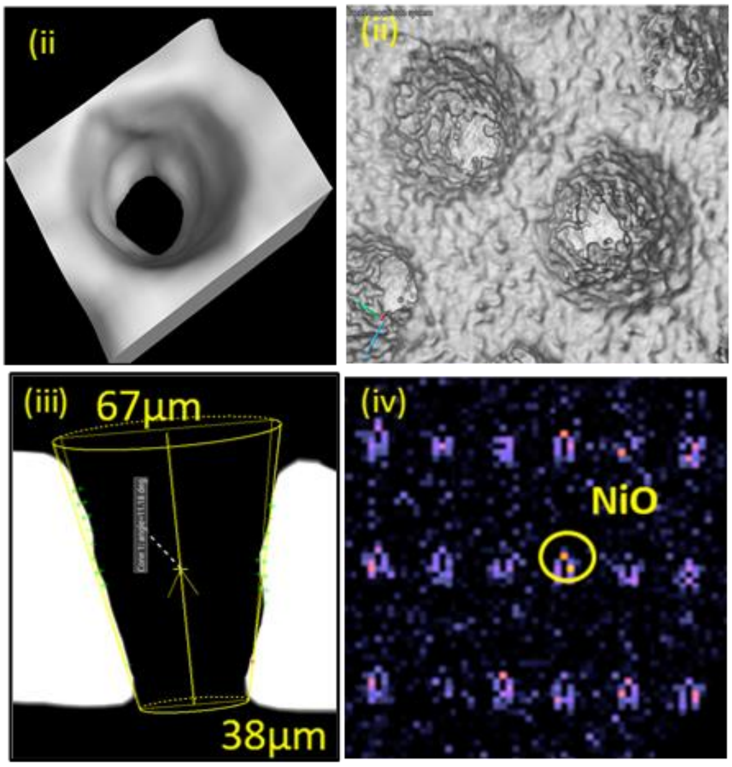

Figure S1. *(a) Magnified view of the patterned hole that has a tapered hole structure and its cross-section (i and ii). Patterned electrode with a depth profile of 67 µm at the top and 38 µm at the bottom (iii).*

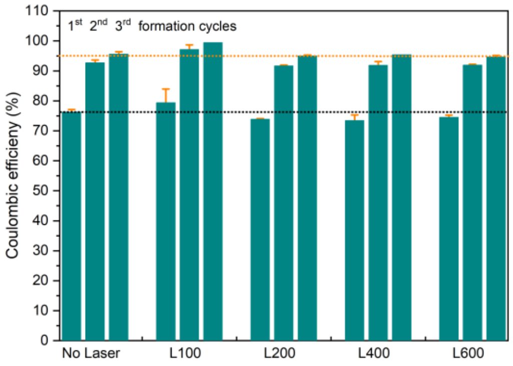

**3rd formation cycle average discharge capacity**		
Electrode	Capacity (mAh)	% loss
No laser	4.03	
L100	3.77	6.4
L200	3.82	5.2
L400	3.96	1.7
L600	4.01	0.5

Figure S2*. Formation cycles coulombic efficiency of pristine and patterned electrodes, error bars depict the variation from two or more cells. Table summarises average capacities of the 3rd formation cycles and the capacity loss from both material loss and laser damage.*

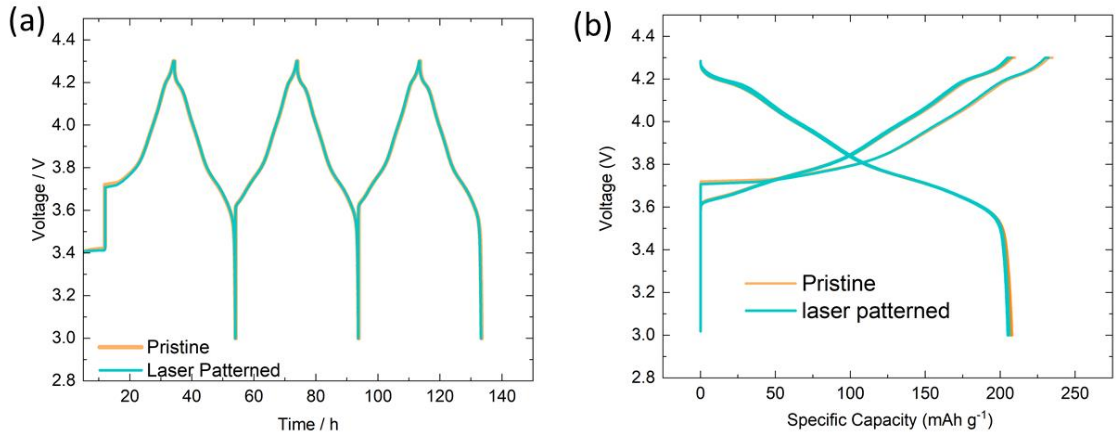

Figure S3. *(a) formation cycles of both pristine and laser patterned electrodes versus lithium metal at C/20 and their corresponding specific capacity (b).*

Table 1: Lithium diffusion coefficients determined from GITT as a function of SOC for pristine and laser-patterned electrodes before and after 50 cycles

Lithium diffusion coefficients obtained from GITT

**Pristine**				**Laser Patterned**			
**Before cycling**		**After cycling**		**Before cycling**		**After cycling**	
**Charge**	**Discharge**	**Charge**	**Discharge**	**Charge**	**Discharge**	**Charge**	**Discharge**
1.77616E-07	1.3966E-07	1.68294E-09	2.13254E-07	1.8895E-09	9.05843E-07	5.23668E-08	4.50239E-07
7.25955E-08	4.7304E-07	1.44985E-09	2.79605E-07	1.79382E-09	6.44927E-07	2.25831E-07	3.19148E-07
8.27618E-08	5.4183E-07	2.42142E-09	2.54208E-06	3.11265E-09	1.99367E-06	3.03294E-07	1.14727E-06
7.71069E-08	4.586E-07	5.10918E-09	3.02842E-06	7.56815E-09	5.04743E-06	2.73868E-07	1.5061E-06
7.35566E-08	3.4152E-07	1.23025E-08	2.06454E-06	1.93567E-08	3.81173E-06	2.38215E-07	1.79307E-06
7.7546E-08	3.3264E-07	2.978E-08	1.54078E-06	6.06298E-08	1.91405E-06	2.4756E-07	1.25511E-06
7.62136E-08	3.517E-07	5.32239E-08	1.43207E-06	1.14835E-07	2.12233E-06	2.75345E-07	1.12132E-06
1.07966E-07	3.0894E-07	6.96159E-08	1.51296E-06	2.13207E-07	1.95507E-06	3.90531E-07	1.02289E-06
1.86554E-07	2.4372E-07	1.07181E-07	1.38737E-06	3.1839E-07	1.65659E-06	7.15963E-07	1.03253E-06
2.58474E-07	1.3995E-07	1.41383E-07	9.20522E-07	3.57614E-07	1.90528E-06	8.64375E-07	7.7234E-07
3.0522E-07	7.7251E-08	2.30547E-07	4.42365E-07	5.3322E-07	1.05889E-06	9.87695E-07	4.65675E-07
3.3132E-07	6.6625E-08	4.87606E-07	3.03991E-07	9.13592E-07	6.24621E-07	1.07671E-06	2.7602E-07
3.48434E-07	6.0724E-08	1.02114E-06	2.14002E-07	1.82388E-06	4.37644E-07	9.17095E-07	2.47991E-07
4.2057E-07	7.6669E-08	1.13328E-06	1.99006E-07	1.55408E-06	5.04021E-07	1.51002E-06	2.03322E-07
4.66002E-07	8.8769E-08	1.23414E-06	2.1105E-07	1.64245E-06	5.25813E-07	1.42726E-06	2.2095E-07
6.01489E-07	8.7002E-08	1.24655E-06	2.28872E-07	1.52955E-06	7.10618E-07	1.83459E-06	2.56472E-07
2.92531E-07		1.10141E-06	2.07616E-07	1.30531E-06	8.16201E-07	6.65486E-07	2.40887E-07
		1.22654E-06	7.76043E-08	1.45758E-06	5.68894E-07	4.16355E-07	1.20849E-07
		1.68539E-06	8.20793E-09	2.0039E-06	4.29194E-08	2.86084E-07	
		5.02737E-07		8.63565E-07	1.11663E-08		
**2.32703E-07**	**2.3679E-07**	**5.14674E-07**	**8.84964E-07**	**7.36276E-07**	**1.36289E-06**	**6.68876E-07**	**6.91788E-07**

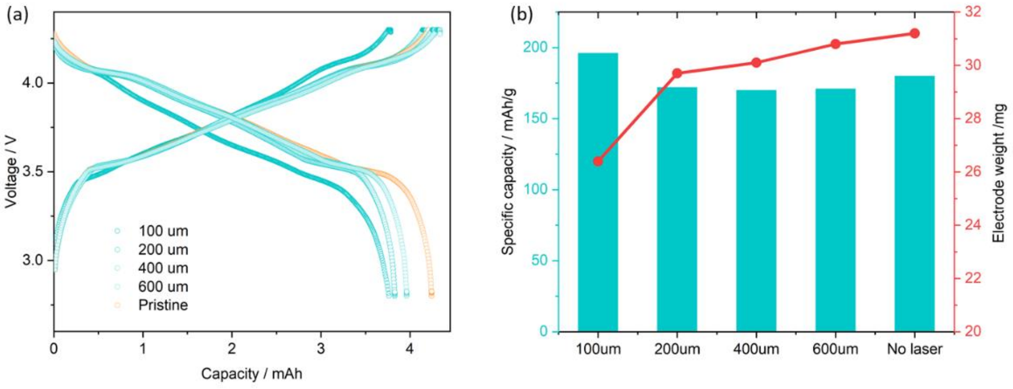

Figure S4. *(a) Formation (3rd) cycle profile of all the electrode investigated and their corresponding specific discharge capacity with weight loss comparison after lasering the electrodes.*

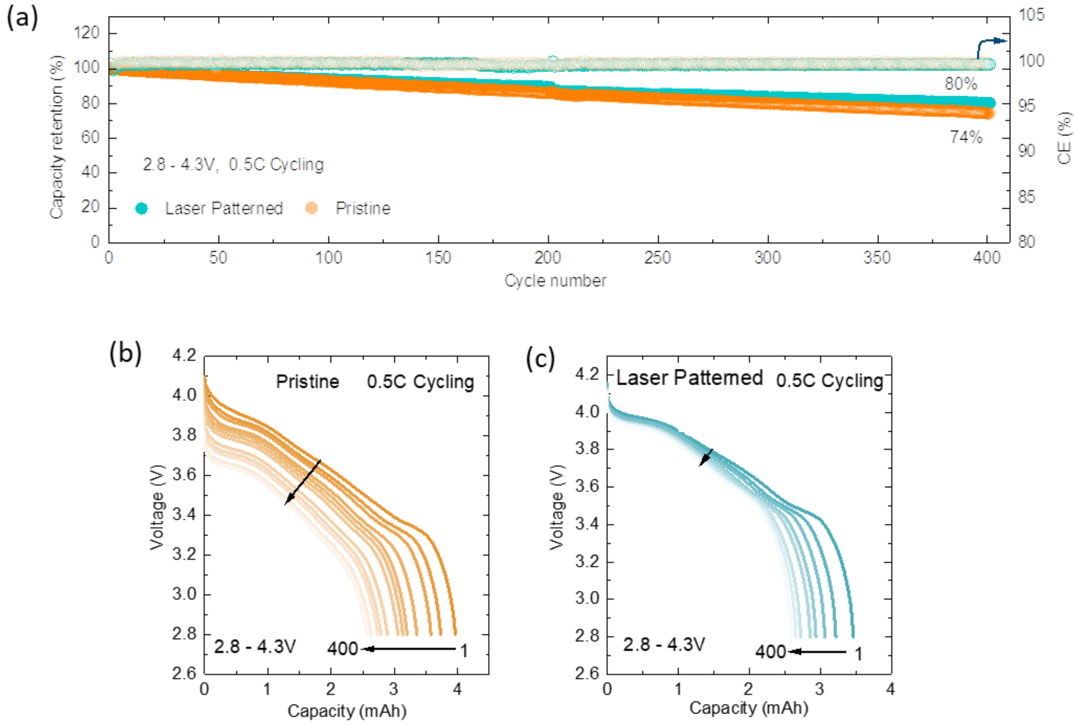

Figure S5*. (a) Full cell cycles of both pristine and laser patterned electrodes versus graphite at C/2 and their capacity /voltage decay over 400 cycles (b and c).*

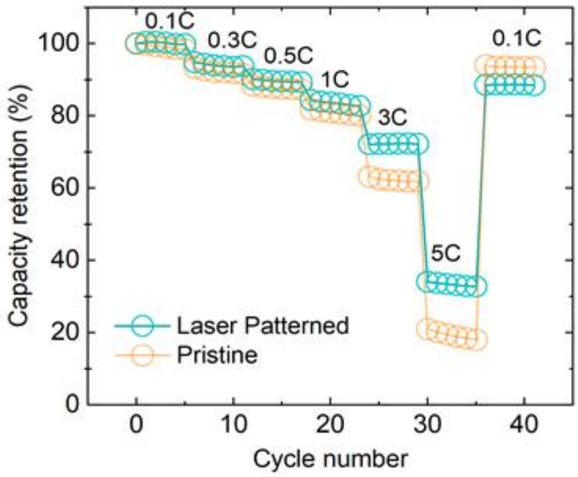

Figure S6. *Rate capability of half cells of patterned and non-patterned electrodes*

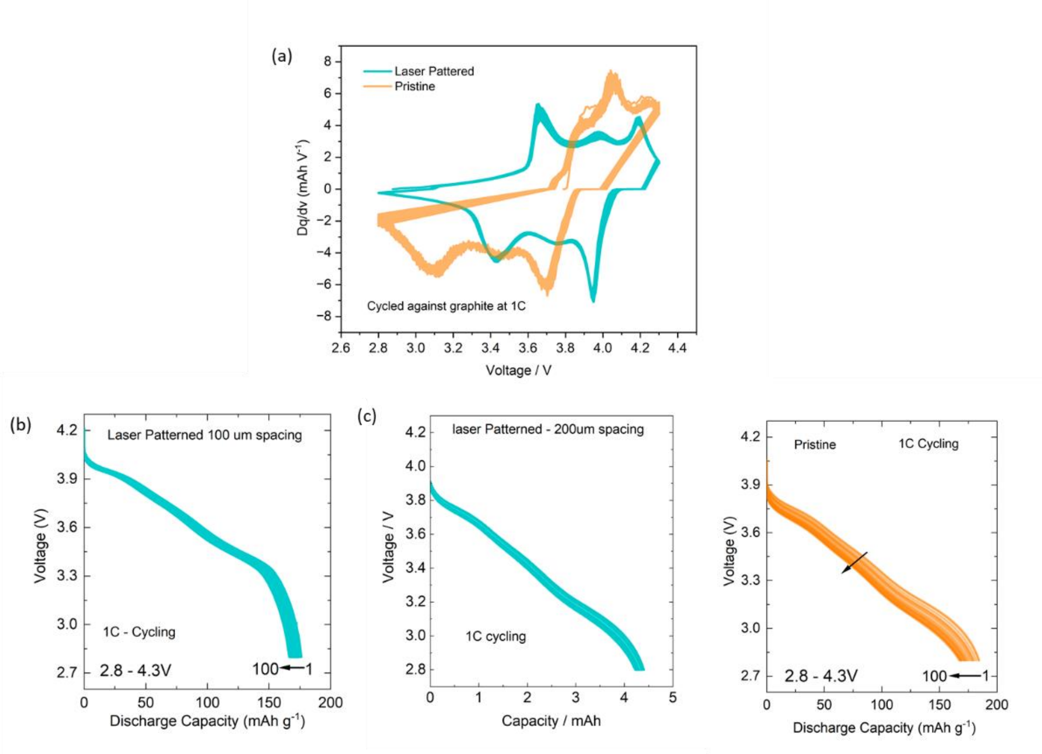

Figure S7. *Full cell cycles of both pristine and laser patterned electrodes versus graphite at 1C over 100 cycles. (a) differential capacity of laser patterned (100um spacing) and pristine electrode, discharge capacities of 100 um spacing (b), 200um spacing (c) lasered electrodes and pristine electrodes (d).*

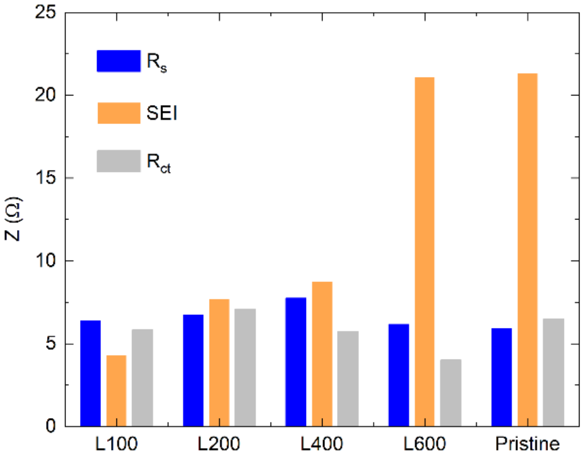

Figure S8. *Rs, Rct and SEI values extracted using Distribution of Relaxation Time (DRT) from the EIS data measured at 50% SoC for different spacings of laser holes.*

Further discussion of charge photometry experiments

As shown in Fig.5 and discussed in the main text, a special electrode design was introduced for charge photometry measurements, where an array of 200 µm-spaced vertical holes pierced through both the NMC layer and aluminium current collector ('through' holes) and an array of holes piercing only the aluminium current collector ('partial' holes). The electrode was assembled in an optically-accessible coin cell with a glass window, oriented such that the NMC was facing the glass window and the current collector was facing the separator, thus enabling optical access to the surface of the active NMC layer (in direct contact with the glass). In this well-defined adapted geometry, electrolyte is able to access the active material layer only via the holes in the current collector. Whilst obtaining charge photometry images, the electrode was abruptly lithiated via a potential step from 4.2 V to 3.0 V, representing an extreme of fast discharging, designed to reveal and spatially-resolve the fundamental kinetic limits of ion transport through the electrode. Fig.5d (main text) shows the normalised optical intensity response at different distances from the holes, divided into radial bands of 20 µm width.

Following the potential step, for all regions, the intensity decreases overall, consistent with previous studies indicating that NMC becomes less optically reflective as it is lithiated[1–3]. For the first ~1.5 min after the potential step, all bands display a very similar initial drop in normalised intensity to ~0.85, as lithiation begins from the electrolyte which is already available throughout the electrode. Close to the 'through' holes (*e.g.* band 1, up to 20 µm from the hole edge), this delithiation then continues rapidly and monotonically, suggesting continual lithiation, with the normalised intensity reaching 0.5 after only 3 minutes. A clear radial dependence is observed, as the lithiation is slower for bands further from the 'through' hole, with band 4 (80-100 µm from the hole centre) achieving half of its intensity change after 32 minutes. This lag of ~29 minutes is indicative of mass transport limitations as lithium-ions diffuse radially from the lasered hole across the electrode.

For the 'partial' holes, a similar radial dependence is observed, with the region furthest from the hole (band 4, 80-100 µm from the hole centre) reaching half of its intensity change ~36 minutes after the region closest to the hole (band 0, directly below the 40 µm-diameter hole in the current collector). A consistent time delay was observed compared to 'through' holes, as discussed in the main text, due to the longer electrolyte diffusion pathway through the electrode thickness. The introduction of vertical holes therefore significantly impacted lithiation rates by providing a direct electrolyte pathway to access the active material furthest from the separator and enabling faster lithiation closer to the hole.

Interestingly, a clear non-monotonic lineshape is observed for the slower bands, including all 'partial' hole bands and 'through' hole bands 3 and 4, which begin to increase again in intensity after ~1.5 mins.

The increasing intensity indicates that the optically-probed surfaces of the active particles are becoming comparatively delithiated (even during overall lithiation of the electrode). This is attributed to lithium redistributing into the core of the solid particles, following the initial lithiation of their surfaces, before a continued supply of lithium-ions can reach them via mass transport from the holes. These bands resume their decrease in intensity (lithiation) after a further ~7 mins for through holes, and ~16 mins for partial holes, indicating that lithium-ion transport within the electrolyte enabled resumed lithiation of these parts of the electrode on longer timescales. This observed intra-particle lithium redistribution underscores the role of local structural features in the interplay between lithium-ion diffusion at different length and timescales in solid NMC particles and in the electrolyte.

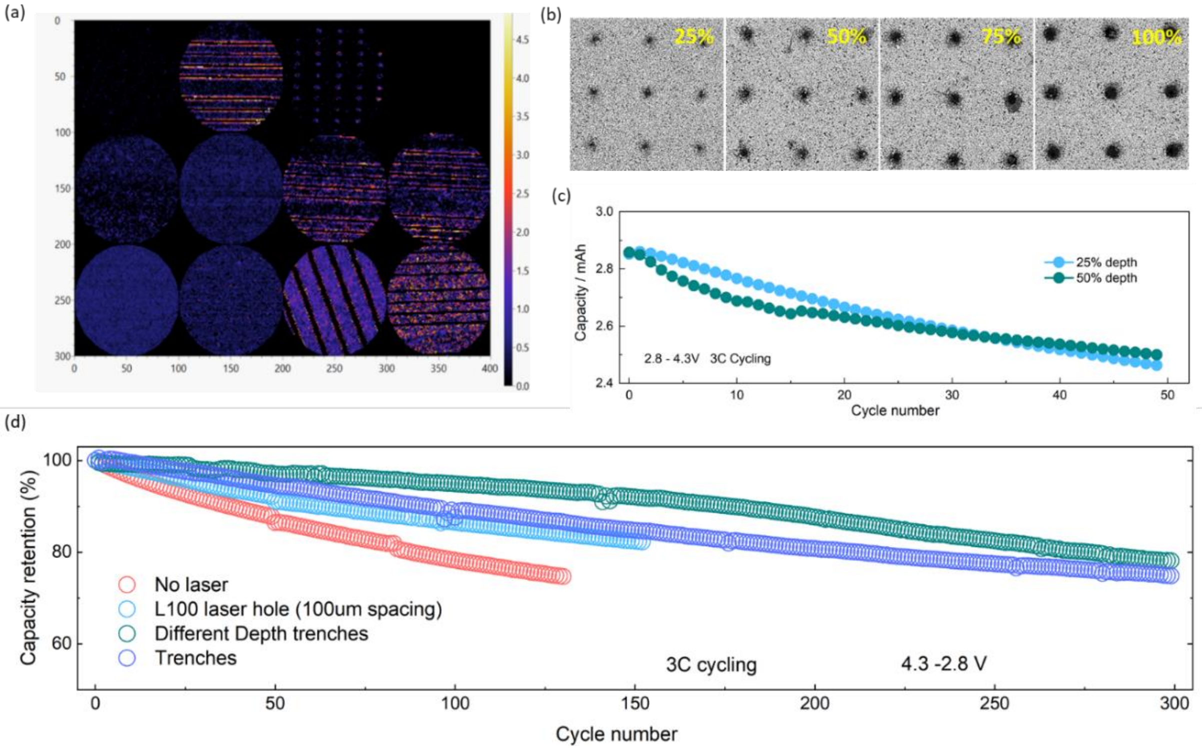

Figure S9. (a) X-ray elemental mapping of laser patterned electrodes, highlighting the spatial distribution and relative intensity of NiO formation induced by laser processing (b) SEM images of laser-drilled hole depths ranging from 25% to 100% of cathode thickness, where 100% depth indicates drilling through the cathode layer without piercing the Al current collector. (c and d) Electrochemical cycling stability of electrodes with different laser-patterned designs, evaluated under a CCCV protocol at 3C cycling.

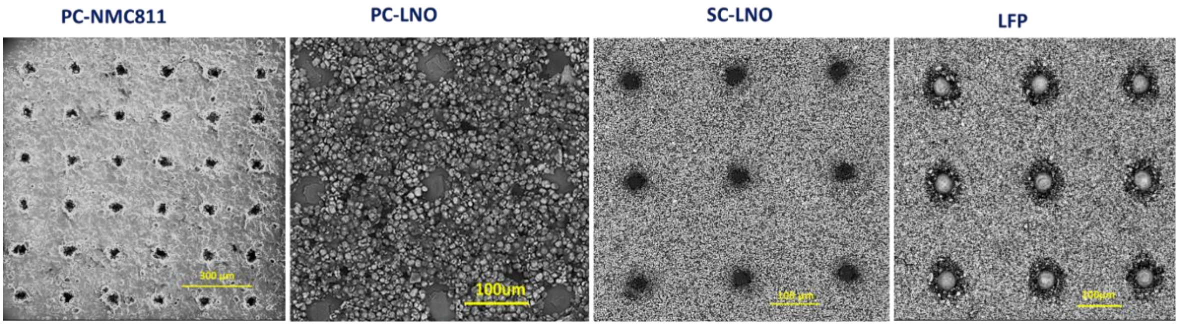

Figure S10. *Demonstration of laser patterning strategy across multiple cathode materials, including polycrystalline NMC811, poly and single crystal LNO, and LFP.*

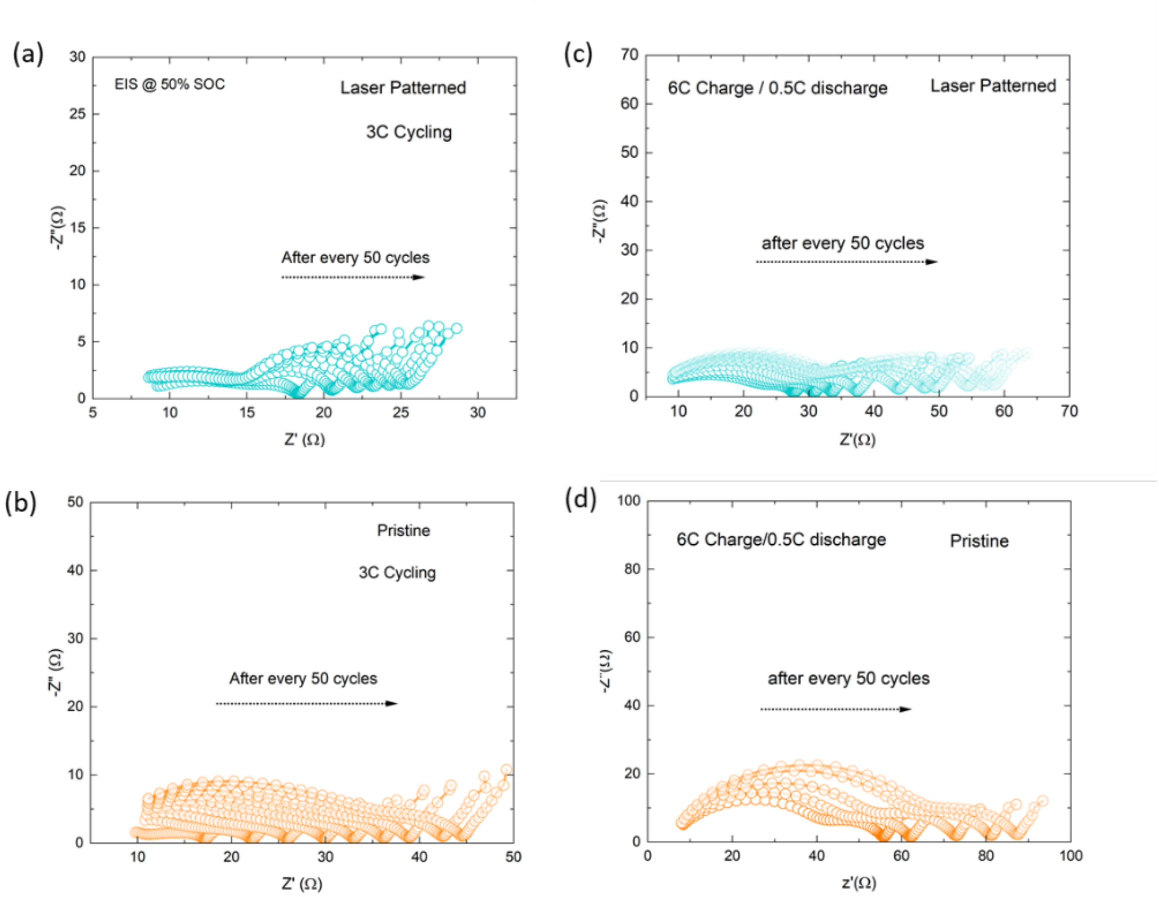

Figure S11. *EIS spectra of both pristine and laser patterned electrodes measured at 50% SOC, laser patterned electrode (100um spacing) cycled at 3C (a), pristine (b). Laser patterned electrode (200um spacing) charged at 6C and 0.5C discharging (a), pristine (b) measured after every 50 cycles.*

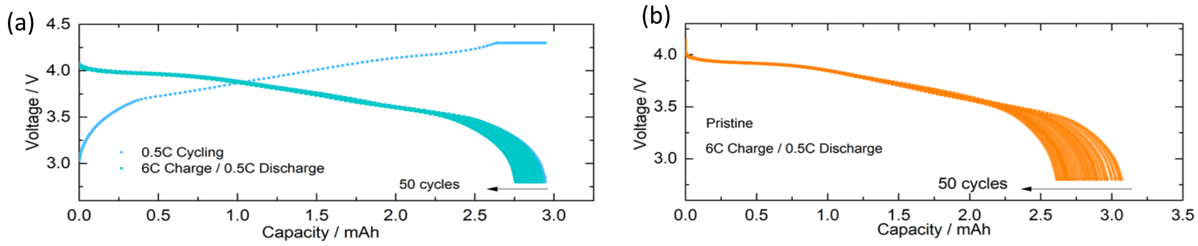

Figure S12. *(a) 0.5C cycling and 6C-charging/0.5C discharging profile of laser patterned and pristine electrodes (b).*

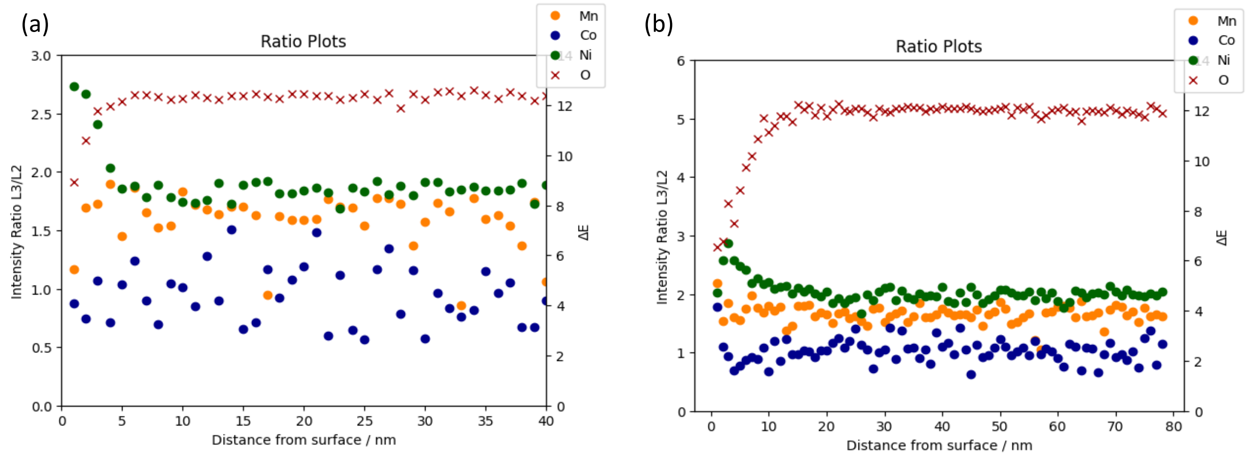

Figure S13. *EELS mapping intensity of the Ni, Co, Mn and O analysed with electrodes cycled at 3C over 400 cycles, lasered electrode (a) and pristine (b).*

References

1. Sharma, N., Vasconcelos, L. S. De, Hassan, S. & Zhao, K. Asynchronous-to-Synchronous Transition of Li Reactions in Solid-Solution Cathodes. *Nano Lett* 22, 5883–5890 (2022).

2. Xu, C. *et al.* Operando visualization of kinetically induced lithium heterogeneities in single-particle layered Ni-rich cathodes. *Joule* 6, 2535–2546 (2022).

3. Lun, Z. *et al.* Operando single-particle imaging reveals that asymmetric ion flux contributes to capacity degradation in aged Ni-rich layered cathodes. *Energy Environ Sci* 18, 4097–4107 (2025).
